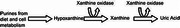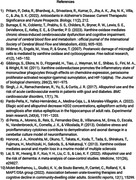# Beyond urate lowering: neuroprotective potential of Allopurinol

**DOI:** 10.1002/alz.093419

**Published:** 2025-01-03

**Authors:** Vyshnavy Balendra

**Affiliations:** ^1^ Saint James School of Medicine, Park Ridge, IL USA

## Abstract

**Background:**

Oxidative stress is formed by a perturbation of redox homeostasis and linked to the development of Alzheimer’s disease (AD) [1]. This imbalance results in an abundance of free radicals that exceeds the antioxidant capacity. Xanthine oxidase (XO) is an enzyme responsible for producing uric acid through the metabolism of purine nucleotides, specifically hypoxanthine and xanthine to uric acid [2]. XO has shown to be upregulated in inflammatory conditions such as AD promoting the generation of free radicals. Thus, allopurinol, an anti‐gout medication, which works to inhibit XO, has a neuroprotective effect and therapeutic potential in delaying neurodegeneration.

**Method:**

A literature search was conducted across scientific, international databases for English, peer‐reviewed articles and reviews published in the last decade using the following terms: allopurinol, xanthine oxidase and Alzheimer’s disease. Focus was placed on animal‐ or human‐based studies. Case reports were excluded.

**Result:**

In‐ vitro studies revealed that elevated XO was associated with increased glial cell proliferation and reduced neuronal survival [3]. Other experimental studies showed XO overexpression in transgenic mice enhanced oxidative stress with increased secretion of pro‐inflammatory cytokines [4]. Allopurinol, an XO inhibitor, has shown to delay neurodegeneration in animal models with AD. Biochemical results in rodents showed decreased oxidative stress (myeloperoxidase levels), mild degenerative impairments in multipolar and bipolar neurons, and decreased apoptotic changes in glial cells after allopurinol treatment [5]. Inhibition of inflammatory molecules reduced vascular damages to the brain and GFAP immunoreactivity in the hippocampus [6]. The drug attenuated microglia infiltration, reduced astrocytes reactivation and prevented axonal loss and demyelination in mouse models [7,8]. Clinically, in population‐based, case‐control studies, allopurinol was associated with a 13‐34% lower risk of AD, lowered cognitive decline, and decreased behavioral symptoms [9,10].

**Conclusion:**

Evidence ‐ based studies reveal allopurinol, a xanthine oxidase inhibitor, to have a neuroprotective potential against Alzheimer’s disease. Based on the current perspective, further research needs to be conducted in a clinical setting.